# 直链淀粉三（3，5-二甲基苯基氨基甲酸酯）涂敷小尺度硅胶制备高效液相色谱微型手性填充柱

**DOI:** 10.3724/SP.J.1123.2025.07016

**Published:** 2026-07-08

**Authors:** Chenggui ZHANG, Yixing LONG, Heng LIU, Yincui SONG, Yuntao LIANG, Liming YUAN

**Affiliations:** 云南师范大学化学化工学院，云南 昆明 650500; School of Chemistry and Chemical Engineering，Yunnan Normal University，Kunming 650500，China

**Keywords:** 高效液相色谱, 微型填充柱, 直链淀粉三（3，5-二甲基苯基氨基甲酸酯）, 硅胶, high performance liquid chromatography, miniature column, amylose tris（3，5-dimethylphenylcarbamate）, silica gel

## Abstract

利用传统手性色谱柱分离手性化合物消耗流动相较多，所需时间较长，并且手性柱中的手性固定相填充量较多且价格昂贵。微型填充柱在提升分离效率、实现分离仪器小型化以及降低固定相、流动相消耗方面表现出广阔的应用前景。本研究将直链淀粉三（3，5-二甲基苯基氨基甲酸酯）（ADMPC）溶液分别涂敷在粒径为500 nm和1.7 μm的硅胶表面上，制备成2种10 mm长的微型填充柱，即ADMPC-1（粒径500 nm硅胶）、ADMPC-2（粒径1.7 μm硅胶）。利用上述两种微型填充柱，采用高效液相色谱法（HPLC）分别成功拆分了23种、20种外消旋体。两种微型填充柱与商品Chiralpak AD-H手性柱具有一定的互补性。该填充柱最突出的优点是分离时间短，分析效率极大提高；所用流动相少，减少了环境污染；同时，也可为今后色谱仪器小型化研究提供所需的小型色谱柱。

## Abstract (en)

The separation of chiral compounds using common dimension chiral chromatographic columns consumes a large of mobile phase and a long time. This is mainly due to the fact that the inner diameter of common chiral chromatographic column is usually 4.6 mm and the column length is 25 cm. The larger column volume requires more mobile phase to drive the sample through the separation process， and the longer column length increases the migration path of the sample within the column， resulting in an extended separation time. Moreover， the common dimension chiral column is filled more stationary phase which is usually expensive. The micro-packed column， due to their structural characteristics， with inner diameters typically controlled and a few millimeters and significantly shortened column lengths， show broad application prospects in improving separation efficiency， achieving miniaturization of separation instruments， and reducing the consumption of stationary and mobile phases. In this study， amylose tris（3，5-dimethylphenylcarbamate） （ADMPC） was used as the chiral selector. Amylose molecules have a helical structure， and the size of the helical cavity and the chiral environment can be regulated through substituent modification. The introduction of 3，5-dimethylphenylcarbamate groups can enhance the hydrophobic interaction， hydrogen bonding， and *π-π* stacking between ADMPC and chiral compounds， thereby improving chiral recognition ability. Due to its excellent selectivity and wide applicability， it has become a research hotspot in the field of chiral separation. Two 10-millimeter-long micro-packed columns were prepared by coating ADMPC on silica gel particles with diameters of 500 nm and 1.7 μm， respectively， and were named ADMPC-1 （500 nm silica gel） and ADMPC-2 （1.7 μm silica gel）. On these two micro-packed columns， 23 and 20 racemates， respectively， including various types such as alcohols， esters， ketones， and acids， achieved good separation effects. Notably， there are certain differences in separation selectivity between these two micro-packed columns， showing a certain degree of complementarity. Moreover， these two micro-packed columns also exhibit this complementary property compared with the commercial Chiralpak AD-H chiral column. The most significant advantage of micro-packed columns is the short separation time， most compounds can be separated within 8 min， and many compounds can achieve baseline separation. Commercial chiral columns， due to their longer column lengths， usually require 15-30 min or even longer for sample retention time， and some compounds may not achieve baseline separation due to insufficient resolution. The shorter column length and optimized small size silica gel of micro-packed columns significantly reduce the retention time of samples and improve resolution， which is of great significance for high-throughput screening experiments （such as chiral compound screening in drug development）， and can significantly improve experimental efficiency. At the same time， due to the reduced column volume， the amount of mobile phase used is also very small. Common dimension chiral columns usually consume tens to hundreds of milliliters of mobile phase for one analysis， while the micro-packed columns in this study only require a few milliliters or even less for one analysis. This greatly reduces the generation of organic waste and lowers environmental pollution， in line with the development concept of green chemistry. In addition， compared with common dimension chiral columns， the amount of stationary phase in micro-packed columns is significantly reduced， significantly lowering the cost； at the same time， it can also provide suitable column dimemsion for the development of miniaturized chromatographic systems.

手性是指一个体系，它的镜像不能通过转动、平移等空间变换而完全一致。当一个系统无法通过任何空间操作与其镜像完全叠加时，称该系统具有手性^［1，2］^。手性拆分是指通过一定方法将外消旋体拆分为单一对映体的过程，在各个学科中都有着非常重要的作用。从传统的结晶拆分法^［3］^、色谱拆分法^［4］^到膜拆分法^［5］^，选择哪种方法取决于分析的具体需求和样品的性质。

高效液相色谱（HPLC）因其分离效率高、应用范围广、快速等优点而被广泛应用于药物分析^［6］^、药物代谢研究^［7］^、临床试验^［8］^、环境监测^［9］^和食品安全^［10］^等领域，是分析分离领域的黄金标准。

几十年来，发展出了许多不同种类的手性固定相（CSP）。例如，Pirkle型^［11］^、配体交换型^［12］^、分子印迹型^［13］^、大环抗生素^［14］^、蛋白质^［15］^、多糖^［16］^、环糊精^［17］^、冠醚^［18］^、环果糖^［19］^以及合成聚合物^［20］^等。此外，金属-有机框架^［21］^、共价有机框架^［22］^、多孔有机笼^［23］^和金属-有机环^［24］^已成为CSP的研究热点。

多糖作为手性选择剂可追溯到1951年，Kotake等^［25］^报道了将纤维素作为手性选择剂在纸色谱中分离对映体。在20世纪70年代初期，由Hesse和Hagel制备的微晶纤维素三乙酸酯（CTA）是第一个被用作手性识别剂的纤维素衍生物^［26］^。此后，Okamoto团队^［27］^进一步优化了多糖衍生物类手性选择剂，制备得到用于HPLC的多糖衍生物类手性固定相。目前直链淀粉三（3，5-二甲基苯基氨基甲酸酯）（ADMPC）是位于前列的被广泛使用的液相色谱手性固定相。

由于近年来手性药物及生理活性物质的不断增多，而通过合成通常很难达到光学纯要求，必须采用手性分离技术进行纯化和检验，因此对新型手性色谱柱技术进行深入研究具有重要意义。利用传统色谱柱分离手性化合物往往较为耗时，但手性选择剂和手性分析物往往较为昂贵，所以小型化分离仪器是分离多种分析物的一个有吸引力的选择。微型填充柱操作更加便利，在缩短分析时间、减少固定相、流动相和样品消耗量的同时实现高通量高效分离，而对样品的分辨率仍能保持较高水平，且对于色谱仪器的小型化可以提供所需的小的色谱柱。目前将多糖微型填充柱应用于手性分离分析还未见文献报道。

本研究将ADMPC分别涂敷在粒径500 nm和1.7 μm的硅胶表面，成功制备出ADMPC-1（粒径500 nm）和ADMPC-2（粒径1.7 μm）两种微型填充柱，并将其应用于HPLC，以实现高速高效的手性拆分。并在3种洗脱液条件下评估上述两种微型填充柱的对映体选择特性。同时，将上述两种微型填充柱与商品Chiralpak AD-H手性柱进行对比。

实验结果表明上述两种微型填充柱均拥有理想的手性拆分效果，其中粒径相对较小的ADMPC-1柱拆分效果更好。在手性分离方面，两种微型填充柱在彼此之间、与商品Chiralpak AD-H手性柱之间都展现出互补性。此外，不同温度、分析物含量和流速对两种微型填充柱手性分离的影响不同，但两种微型填充柱的稳定性均较好。

## 1 实验部分

### 1.1 仪器、试剂和材料

DRX-500核磁共振波谱仪（NMR）、傅里叶红外光谱仪（FT-IR）（德国Bruker公司）；P230 Ⅱ高压恒流泵、UV 230 Ⅱ紫外检测器、AT-330柱温箱（大连Elite分析仪器有限公司）；1666高效液相色谱装柱机（美国Alltech有限公司）；RE52CS-1旋转蒸发器、B-260恒温水浴锅（中国上海亚荣生化仪器厂）。

粒径分别为500 nm、1.7 μm的硅胶、二乙胺、三氟乙酸购于上海Macklin生化科技股份有限公司；正己烷、异丙醇、甲醇购于天津市风船化学试剂有限公司；*N，N*-二甲基甲酰胺（DMF）购于成都科隆化学有限公司；直链淀粉、3，5-二甲基苯基异氰酸酯购于上海Adamas-beta试剂有限公司。实验中所用到的*N，N*-二甲基甲酰胺的纯度为99.5%，其余试剂的纯度均为99.0%。

### 1.2 ADMPC的制备

ADMPC参照文献［^1^］合成。称取500 mg直链淀粉置于烧瓶中，在氮气气氛下加入20 mL无水吡啶充分搅拌后，加入3，5-二甲基苯基异氰酸酯2.0 mL，100 ℃下回流24 h。向其中加入大量甲醇后收集白色沉淀，在65 ℃下进行5 h的真空干燥，最后获得产物直链淀粉（3，5-二甲基苯基氨基甲酸酯）。其合成机理如图1所示。

**图1:**
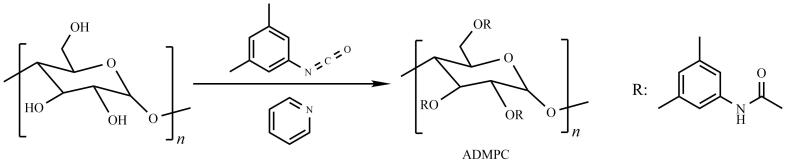
直链淀粉三（3，5-二甲基苯基氨基甲酸酯）的合成

### 1.3 ADMPC涂敷硅胶固定相的制备

取2份质量为0.12 g的ADMPC，分别溶于3.0 mL *N*，*N*-二甲基甲酰胺中。接着，依次取0.48 g粒径分别为500 nm和1.7 μm的硅胶，分别置于2个圆底烧瓶中，然后将上述溶有ADMPC的*N，N*-二甲基甲酰胺溶液分别缓慢匀速地倒入不同的上述圆底烧瓶中，然后在旋转蒸发器上，在溶剂真空挥发后，使其涂敷到不同粒径的硅胶表面上，制备得到涂敷有直链淀粉三（3，5-二甲基苯基氨基甲酸酯）的硅胶固定相。

### 1.4 ADMPC涂敷硅胶微型填充柱的制备

使用匀浆填充方法，填充尺寸为（10 mm×4.6 mm）的不锈钢空柱。将涂敷有直链淀粉三（3，5-二甲基苯基氨基甲酸酯）的两种硅胶固定相，分别悬浮在正己烷-异丙醇（90∶10，*V/V*）溶液中，制备固定相匀浆液。接着，将它们分别注入匀浆槽，并以正己烷-异丙醇（90∶10，*V/V*）溶液作置换溶剂。通过施加30 MPa的压力来填充色谱柱，泄压后将柱子取下。在测样前，用流动相以0.050 mL/min的流速冲洗柱子1~2 h。

### 1.5 色谱拆分条件及性能评价参数

流动相：正己烷-异丙醇（90∶10，*V/V*）、正己烷-异丙醇-三氟乙酸（90∶10∶0.2，*V/V/V*）、正己烷-异丙醇-二乙胺（90∶10∶0.2，*V/V/V*）；柱温：25 ℃；手性化合物浓度：1.0 mg/mL；进样体积：2.0 μL；检测波长：230~270 nm；ADMPC-1微型填充柱流速：0.050 mL/min，ADMPC-2微型填充柱流速：0.040 mL/min。进样体积变化范围：2.0~10 μL，梯度2.0 μL；温度变化范围：20~40 ℃，梯度5.0 ℃；稳定性进样次数：50~250次，梯度50次；流速变化范围：0.01~0.11 mL/min。性能探究过程中，ADMPC-1使用1-（4-氟苯基）乙醇，ADMPC-2使用1-苯基乙醇。

## 2 结果与讨论

### 2.1 ADMPC涂敷硅胶固定相的表征

#### 2.1.1 核磁共振和傅里叶红外光谱表征

对直链淀粉三（3，5-二甲基苯基氨基甲酸酯）进行核磁共振氢谱（^1^H NMR）表征，^1^H NMR （500 MHz，C_5_D_5_N）直链淀粉2、3、6-位引入的3，5-二甲基苯基氨基甲酸酯上的甲基质子化学位移为1.60~2.20和2.20~2.40，直链淀粉上质子的化学位移为4.0~6.0，苯环上质子吸收峰的化学位移为6.30~7.10，氨基的化学位移为9.10~9.80，且峰的积分面积与对应的质子个数相符，由此可知，成功制得了直链淀粉三（3，5-二甲基苯基氨基甲酸酯）。

图2所示为ADMPC的红外光谱图，在3 316 cm^-1^处观察到R-NH-R′的N-H伸缩振动吸收峰，2 915 cm^-1^处的吸收峰来源于烷基的不对称伸缩振动，在1 736 cm^-1^处检测到C=O的强吸收峰，C-O-C伸缩振动吸收峰在1 220 cm^-1^处。结合ADMPC的^1^H NMR表征，有力证明了ADMPC的成功合成。

**图2:**
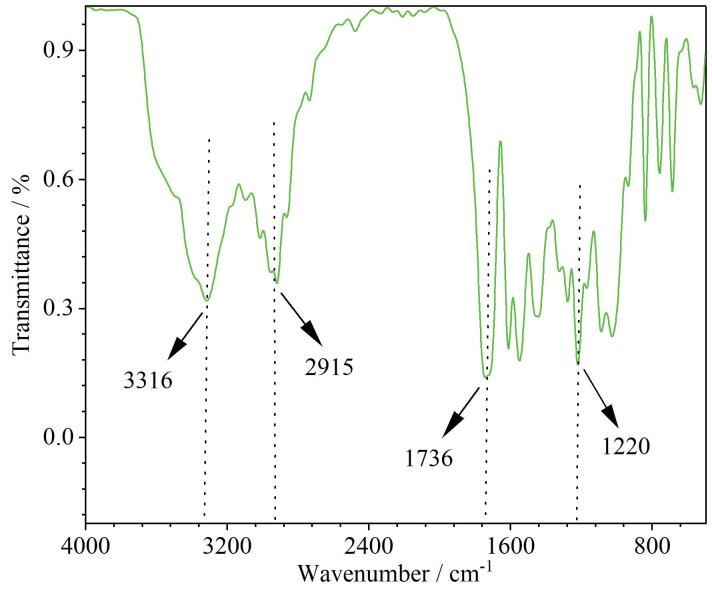
ADMPC的傅里叶红外光谱图

#### 2.1.2 扫描电镜表征

图3为粒径为500 nm的裸露非晶硅胶形貌图（图3a）和ADMPC涂敷于粒径为500 nm的硅胶形貌图（图3b）。可以看出，粒径为500 nm的裸露硅胶为分散的无定形结构；500 nm硅胶由裸露分散的无定形硅胶聚集在一起，表明ADMPC已经成功涂敷在500 nm硅胶上。

**图3:**
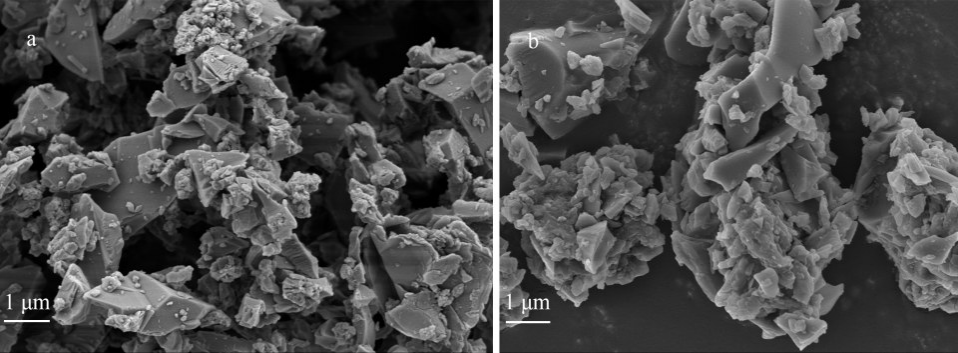
（a）粒径为500 nm裸露非晶硅胶和（b）ADMPC-1固定相的SEM图

图4为粒径1.7 μm的裸露球形硅胶形貌图（图4a）和ADMPC涂敷在粒径为1.7 μm的球形硅胶形貌图（图4b）。可以看出，粒径1.7 μm的裸露硅胶为相对光滑的球形结构；硅胶表面包裹上一层厚厚的物质，表明ADMPC成功涂敷到粒径为1.7 μm的硅胶表面。

**图4:**
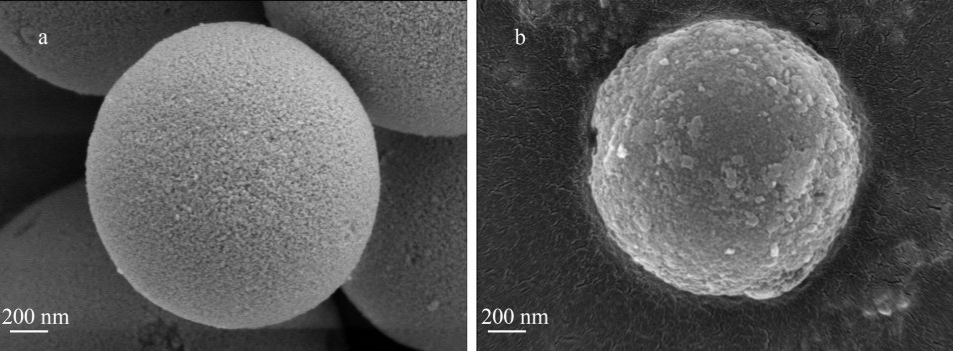
（a）粒径为1.7 μm裸露球形硅胶和（b）ADMPC-2固定相的SEM图

### 2.2 微型填充柱在正相（NP）洗脱下对外消旋体的拆分

本实验对ADMPC-1微型填充柱、ADMPC-2微型填充柱的拆分能力进行评价，两种不同的微型填充柱分离外消旋体的分离数据见表1。ADMPC-1微型填充柱对23种外消旋体有不同程度的拆分，拆分色谱图如图5所示。有20种外消旋体在ADMPC-2微型填充柱上得到分离，拆分色谱图如图6所示，上述外消旋体的拆分结果表明ADMPC-1微型填充柱、ADMPC-2两种微型填充柱之间具有一定的互补性。综上对比分析可知，ADMPC-1微型填充柱的拆分能力更强，拆分效果更好，也就是说，当柱长和内径相同时，随着硅胶粒径的减小，分离效果逐渐增强。这是因为较小的粒径可以减少溶质分子在柱内的纵向和涡流扩散，填充较小尺寸颗粒的色谱柱孔隙较少，最终可以通过降低塔板高度（*H*）来提高色谱柱效率。

**表 1:** NP洗脱模式下手性化合物在ADMPC-1、ADMPC-2和Chiralpak AD-H柱上的分离数据

Racemate	ADMPC-1			ADMPC-2			Chiralpak AD-H		
	*k*_1_	*α*	*R*_s_	*k*_1_	*α*	*R*_s_	*k*_1_	*α*	*R*_s_
1-（4-Chloro）phenylethanol	2.06	2.20	1.17	2.67	1.00	-	0.86	1.04	0.49
Warfarin	6.71	2.34	1.06	9.83	1.79	1.38	0.55	1.89	2.31
Ibuprofen^a）^	2.07	1.68	0.85	2.86	1.00	-	0.71	1.00	-
3-Benzyloxy-1，2-propanediol	2.12	1.91	1.02	1.85	1.00	-	2.24	1.04	0.70
Bupropion hydrochloride	2.10	1.84	1.22	3.77	1.81	1.03	1.93	1.00	-
4-Chlorodiphenylmethanol	2.09	3.2	1.67	2.78	1.94	1.31	1.65	1.14	2.01
Cetirizine^b）^	1.76	1.91	1.08	1.88	1.00	-	1.42	1.26	2.73
1-Phenylethanol	2.02	1.88	1.09	2.94	1.79	1.34	0.83	1.00	-
1-Phenyl-1-butanol	2.06	1.93	1.05	2.86	1.54	0.73	0.75	1.00	-
1-Phenyl-1-pentanol	2.07	1.61	0.61	2.89	1.00	-	0.73	1.00	-
4-Phenyl-2-butanol	2.22	1.85	0.91	3.1	1.68	1.17	0.71	1.09	0.84
4-Methyl-1-phenyl-2-pentanol	2.07	1.63	0.71	2.96	1.00	-	0.58	1.00	-
1-（4-Methylphenyl）ethanol	2.02	2.15	1.05	2.81	1.00	-	0.86	1.11	1.16
2-Methoxy-2-phenylethanol	2.01	1.64	0.82	2.34	1.00	-	0.91	1.23	2.70
1-（4-Fluorophenyl）ethanol	2.02	2.12	1.24	2.82	1.99	1.25	0.83	1.00	-
1-Indendanol	2.42	1.00	-	2.87	1.95	1.23	1.19	1.08	0.62
4，4'-Dimethylbenzoin	2.07	1.9	1.30	2.67	1.00	-	5.09	1.09	1.43
4-Methyldiphenylmethanol	2.07	1.00	-	2.86	1.8	1.05	1.65	1.14	1.48
1，2-Bis（4-fluorophenyl）-2-hydroxyacetophenone	2.04	2.14	1.42	4.53	1.35	0.56	4.36	1.10	2.09
Methyl mandelinate	2.04	1.00	-	2.72	2.55	1.58	1.92	1.08	1.56
Ethyl mandelinate	2.01	1.00	-	2.74	2.16	1.31	1.66	1.12	1.80
1-Phenyl-2-propanol	2.05	2.04	1.06	2.79	1.81	1.07	0.78	1.11	0.39
4-Bromo-*α*-methylbenzyl alcohol	2.10	2.31	1.18	2.89	1.95	1.36	1.07	1.05	0.27
4-Fluoro-*α*-methylbenzylamine	2.19	1.91	1.25	2.36	1.00	-	0.62	1.29	2.51
1-（2-Fluorophenyl）ethanol	2.05	2.03	1.12	2.77	1.72	0.97	0.89	1.05	0.23
1-（2-Methylphenyl）ethanol	2.06	2.02	1.25	2.78	1.72	1.20	0.69	1.18	1.22
1-（3-Bromophenyl）ethanol	2.12	1.00	-	2.92	1.79	1.11	1.04	1.00	-
2-Phenylpropionic acid	2.27	1.00	-	2.92	1.47	0.52	0.93	1.22	0.42
1-（3-Fluorophenyl）ethanol	2.05	2.09	1.02	2.54	1.97	1.23	0.86	1.00	-
Fipronil	2.03	1.00	-	2.83	1.94	1.18	0.54	1.00	-

Mobile phases：a）*n*-hexane-isopropanol-trifluoroacetic acid （90∶10∶0.2， *V/V/V*）； b） *n*-hexane-isopropanol-diethylamine （90∶10∶0.2， *V/V/V* ）； -： inseparable； *k*_1_： retention factor of the first enantiomer； *α*： separation factor； *R*_s_： resolution.

**图5:**
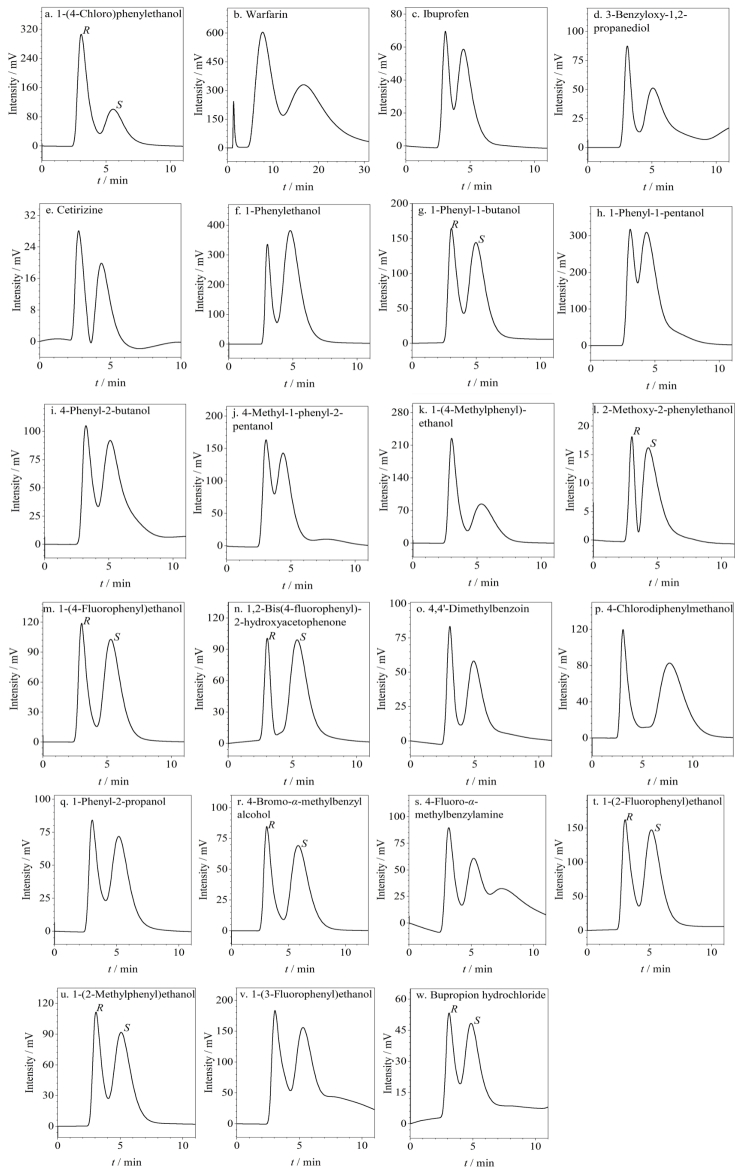
ADMPC-1微型填充柱分离手性化合物的色谱图

**图6:**
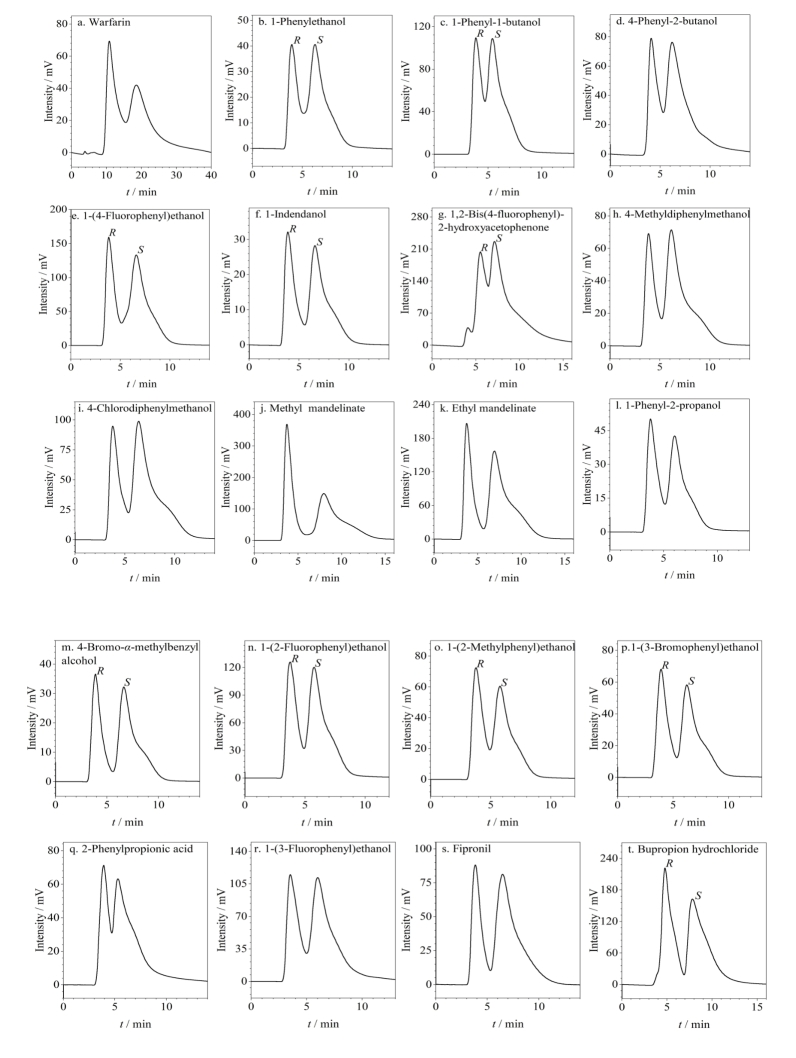
ADMPC-2微型填充柱分离手性化合物的色谱图

此外，在上述两种微型填充柱上得到拆分的手性化合物被作为新的分析物在商品Chiralpak AD-H手性柱上进行分离。由表1可知，部分手性化合物在ADMPC-1微型填充柱、ADMPC-2微型填充柱上得到分离，但是在商品Chiralpak AD-H手性柱上却无法分离。也有一些手性物质在商品化的Chiralpak AD-H型手性柱上实现了基线分离，但在两种微型填充柱上的分离效率较低，甚至不能实现分离。结果表明，ADMPC-1微型填充柱、ADMPC-2微型填充柱与商品Chiralpak AD-H手性柱之间具有良好的互补作用。

### 2.3 进样体积对ADMPC涂敷硅胶制备的微型填充柱手性拆分的影响

#### 2.3.1 ADMPC-1微型填充柱

如图7所示，当1-（4-氟苯基）乙醇的进样体积从2.0 μL增加到10 μL，其对映异构体峰高和峰面积随之增加，保留时间随着进样体积的增加几乎不发生改变，但分辨率下降。由此得出，进样体积过大不利于ADMPC-1微型填充柱进行手性拆分。

**图7:**
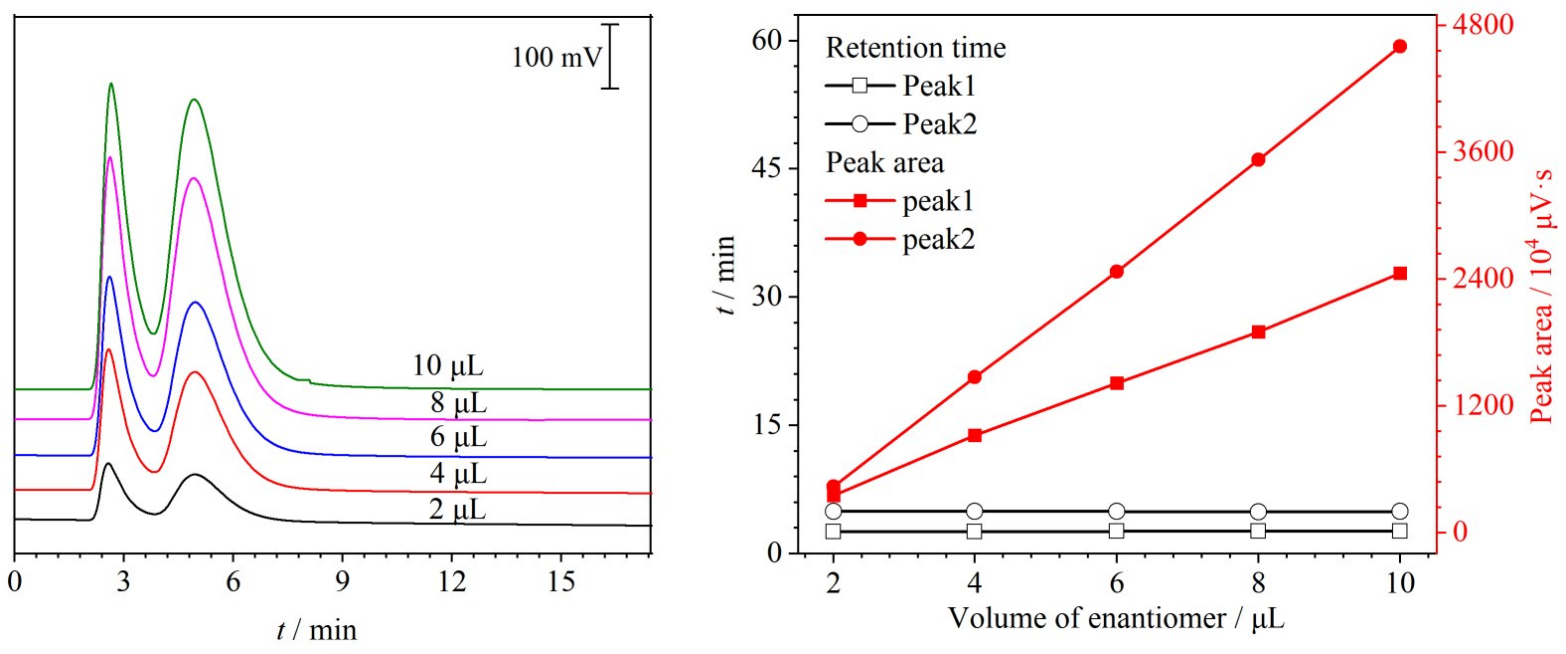
ADMPC-1微型填充柱分离1-（4-氟苯基）乙醇时进样体积对分离效果的影响

#### 2.3.2 ADMPC-2微型填充柱

如图8所示，可知随着进样体积的增加，1-苯基乙醇的保留时间基本不变，峰面积和峰高增加，分离度减小。因此进样体积过大不利于ADMPC-2微型填充柱对外消旋体的拆分。

**图8:**
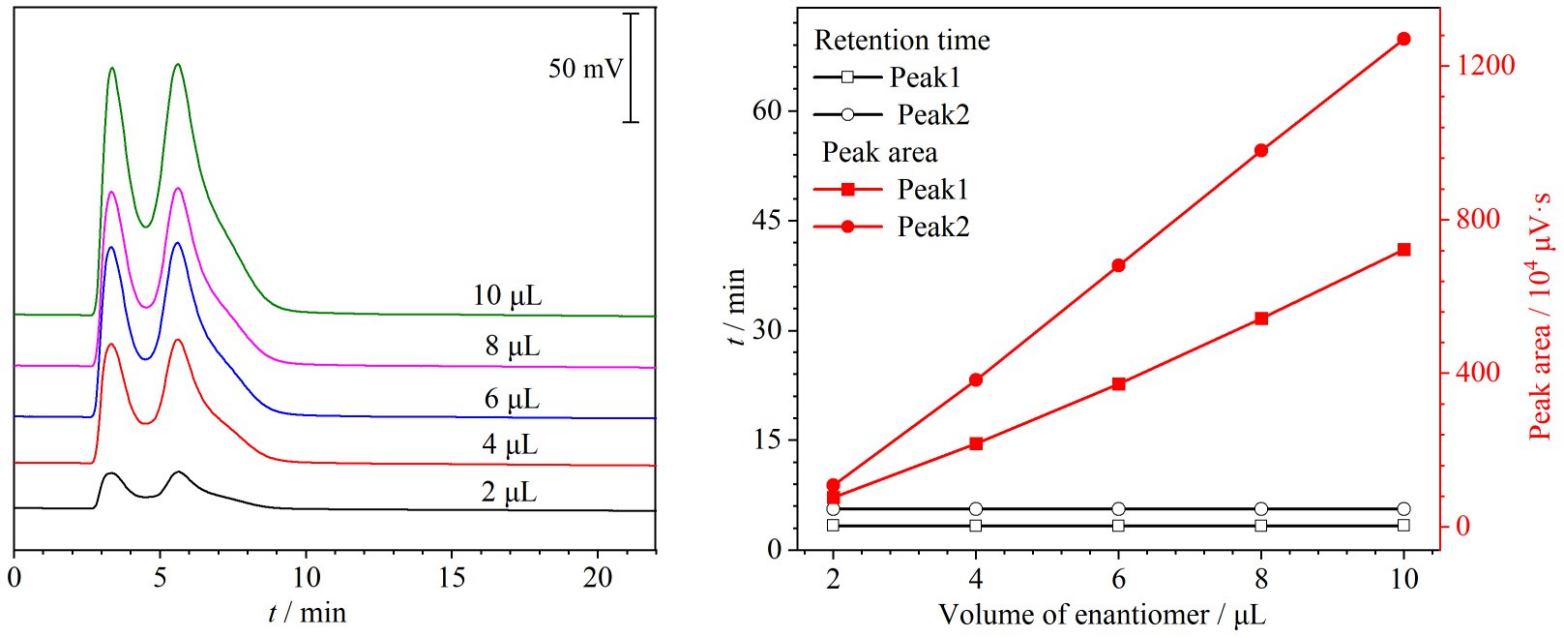
ADMPC-2微型填充柱分离1-苯基乙醇时进样体积对分离效果的影响

### 2.4 温度对ADMPC涂敷硅胶制备的微型填充柱手性拆分的影响

#### 2.4.1 ADMPC-1微型填充柱

实验探索了不同的色谱柱温度对ADMPC-1微型填充柱拆分性能的影响。如图9所示，随着填充柱温度从20 ℃升高到40 ℃，分析物与固定相之间的相互作用减弱，导致其分离度下降。因此，只有在适当的温度下ADMPC-1微型填充柱才能表现出最佳的拆分能力。

**图9:**
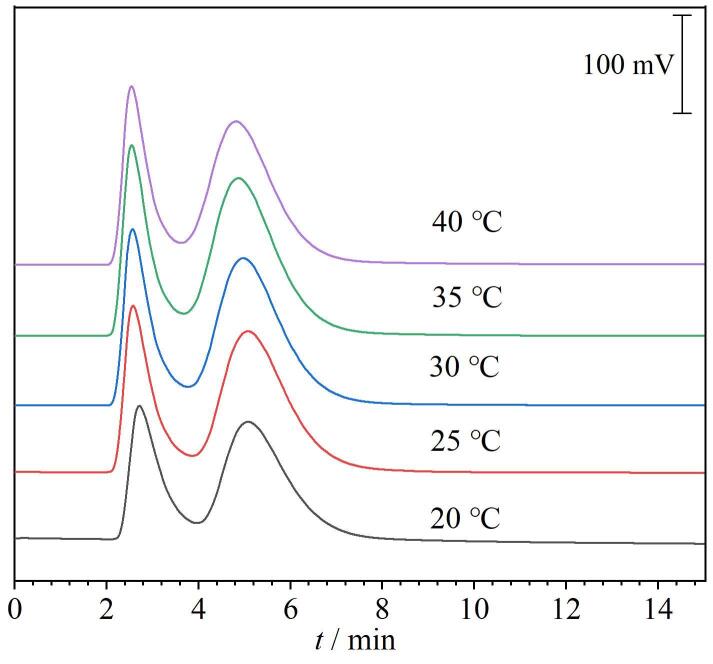
ADMPC-1微型填充柱分离1-（4-氟苯基）乙醇时温度对分离效果的影响

#### 2.4.2 ADMPC-2微型填充柱

探索了不同温度条件下，ADMPC-2固定相与分析物之间的相互作用。如图10所示，测试了1-苯基乙醇在不同柱温下的保留行为，发现适当的升高柱温，1-苯基乙醇的保留时间几乎不变，这是因为在该条件下，分子水平上的分析物与固定相作用力变化较小。而升到一定的温度后，待测物的滞留时间会随着温度的增加而减小，这可能是当温度上升时，分子水平上的分析物与固定相作用力减弱，使其可快速迁移通过色谱柱。

**图10:**
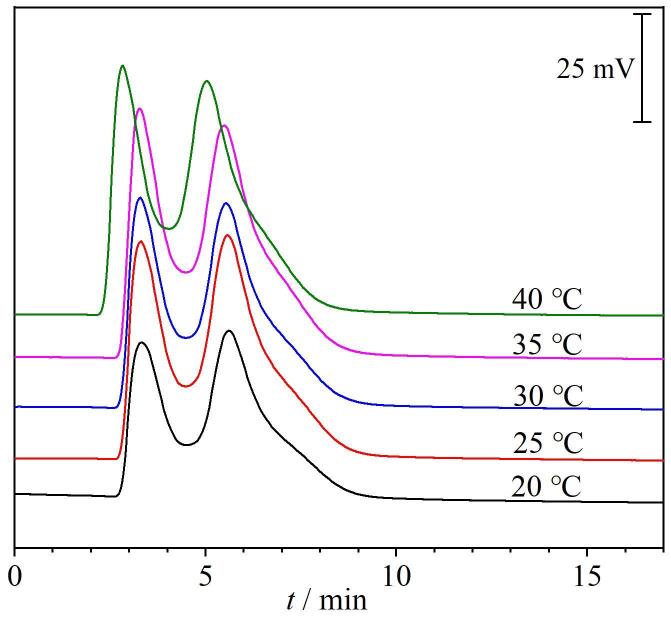
ADMPC-2微型填充柱分离1-苯基乙醇时温度对分离效果的影响

### 2.5 ADMPC涂敷硅胶制备的微型填充柱的稳定性

#### 2.5.1 ADMPC-1微型填充柱

由图11中可以清楚看到，ADMPC-1微型填充柱对1-（4-氟苯基）乙醇的拆分效果基本不变，保留时间和峰面积的RSD分别小于0.93%和1.83%。结果表明，ADMPC-1微型填充柱具有良好的稳定性。

**图11:**
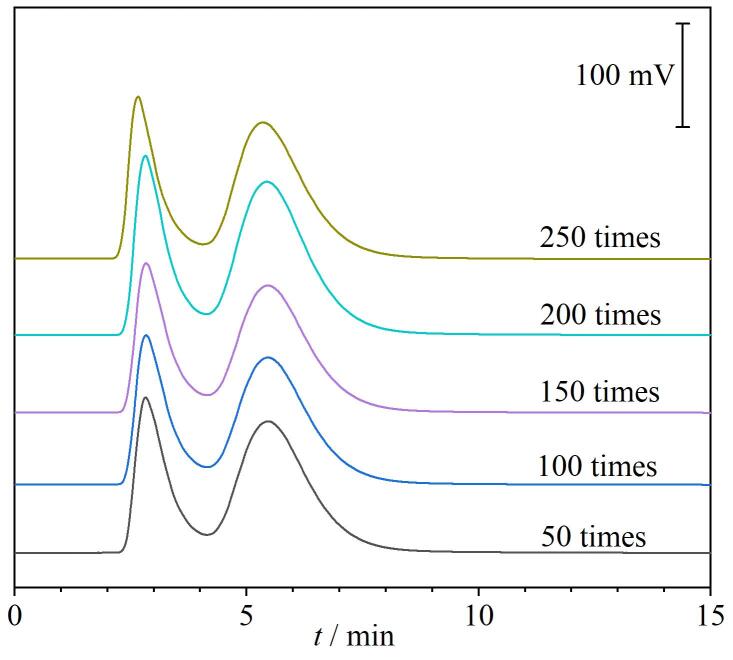
ADMPC-1微型填充柱分离1-（4-氟苯基）乙醇时的稳定性

#### 2.5.2 ADMPC-2微型填充柱

为研究ADMPC-2微型填充柱的稳定性，我们在相同色谱条件下进行实验。由图12可见，1-苯基乙醇的所有色谱峰均呈现出可重复的色谱分离行为。1-苯基乙醇的保留时间和峰面积的RSD分别小于0.50%和1.38%，表明ADMPC-2微型填充柱具有良好的稳定性。

**图12:**
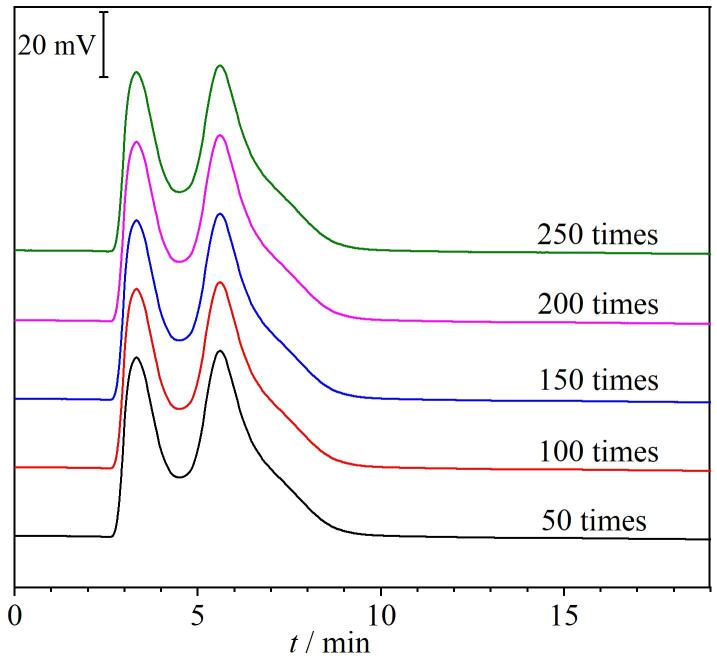
ADMPC-2微型填充柱分离1-苯基乙醇时的稳定性

### 2.6 流速对ADMPC涂敷硅胶制备的微型填充柱手性拆分的影响

Van Deemter曲线图可以定量描述色谱过程的动力学特性，通过分析*H*与线速度的关系，了解色谱柱在不同流速下的性能。本文根据测量的色谱参数计算*H*，并将其与线速度（*u*）作图。如图13所示，在测量的流速范围内获得了ADMPC-1微型填充柱、ADMPC-2微型填充柱的van Deemter曲线图，了解到ADMPC-1微型填充柱在流速为0.050 0 mL/min，即0.050 2 mm/s时获得最低*H*，ADMPC-2微型填充柱在流速为0.040 0 mL/min，即0.040 1 mm/s时获得最低*H*。本文不同粒径的硅胶，分辨率不同，*H*不同，最佳线速度也不相同。由图13可清楚看到，粒径越小*H*越小，最佳线速度越大，柱效越好。因为*H*是柱效率的一个衡量标准，提供了评估柱性能的方法，较小的*H*对应较好的柱性能。

**图13:**
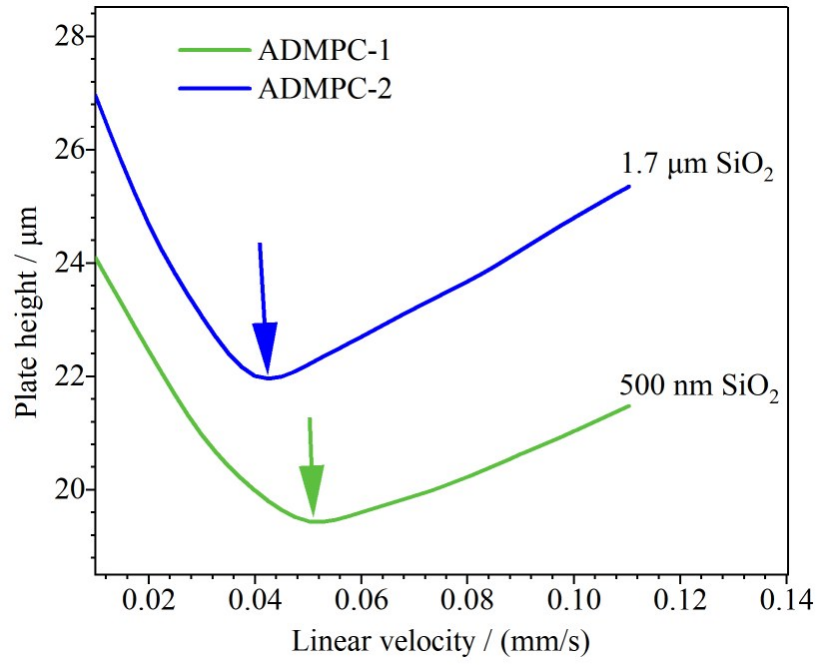
塔板高度与线速度关系图

## 3 结论

本研究将两种不同粒径的硅胶用作ADMPC的载体制备固定相，用于HPLC的手性化合物分离。通过将合成的ADMPC和两种粒径的硅胶设计出ADMPC-1、ADMPC-2两种微型填充柱，所得的两种微型填充柱对小分子醇类、脂类、酮类手性化合物表现出优异的分离性能。可能是因为手性微环境、*π*-*π*相互作用以及氢键的协同效应在微型填充柱的HPLC体系中发挥着重要作用。与商品Chiralpak AD-H手性柱相比，ADMPC-1、ADMPC-2两种微型填充柱仍具有高分离效率和良好分辨率。此外，ADMPC-1、ADMPC-2两种微型填充柱具有良好的色谱稳定性。本文将ADMPC涂敷在小粒径硅胶上制备微型填充柱用于手性HPLC分离分析，为手性色谱分离提供了新思路。
